# Oridonin Protects against Myocardial Ischemia–Reperfusion Injury by Inhibiting GSDMD-Mediated Pyroptosis

**DOI:** 10.3390/genes13112133

**Published:** 2022-11-17

**Authors:** Jiahui Lin, Xianhui Lai, Xiaoxi Fan, Bozhi Ye, Lingfeng Zhong, Yucong Zhang, Ruiyin Shao, Si Shi, Weijian Huang, Lan Su, Miaomiao Ying

**Affiliations:** 1 First School of Medicine, Wenzhou Medical University, Wenzhou 325000, China; 2 Department of Cardiology, Yuhuan County People’s Hospital of Zhejiang Province, Taizhou 318000, China; 3 Key Laboratory of Cardiovascular Disease, Department of Cardiology, First Affiliated Hospital of Wenzhou Medical University, Wenzhou 325000, China; 4 Department of Pathology, First Affiliated Hospital of Wenzhou Medical University, Wenzhou 325000, China

**Keywords:** oridonin, cardiac ischemia/reperfusion injury, pyroptosis, GSDMD

## Abstract

Pyroptosis serves a crucial function in various types of ischemia and reperfusion injuries. Oridonin, a tetracycline diterpene derived from *Rabdosia rubescens*, can significantly inhibit the aggregation of NLRP3-mediated inflammasome. This experiment is aimed at investigating the effect of oridonin on pyroptosis in mice cardiomyocytes. Based on the models of myocardial ischemia/reperfusion (I/R) and hypoxia/reoxygenation (H/R), Evans Blue/TTC double staining, TUNEL staining, and Western blotting were applied to determine the effects of oridonin on myocardial damage, cellular activity and signaling pathways involved in pyroptosis. During I/R and H/R treatments, the extent of gasdermin D-N domains was upregulated in cardiomyocytes. Apart from that, oridonin improved cell survival in vitro and decreased the myocardial infarct size in vivo by also downregulating the activation of pyroptosis. Finally, the expression levels of ASC, NLRP3 and p-p65 were markedly upregulated in cardiomyocytes after H/R treatment, whereas oridonin suppressed the expression of these proteins. The present experiment revealed that myocardial I/R injury and pyroptosis can be alleviated and inhibited by oridonin pretreatment via NF-κB/NLRP3 signaling pathway, both in vivo and in vitro. Therefore, oridonin may serve as a potentially novel agent for the clinical treatment of myocardial ischemia-reperfusion injuries.

## 1. Introduction

With its high morbidity and mortality rate, acute myocardial infarction (MI) has become a major contributor to death and disability around the world. Reports show that there are approximately three million patients who are diagnosed with this disorder annually, thus leading to a huge strain on the economy and the normal functioning of society [1]. For patients with myocardial infarction, the most effective therapeutic intervention is primary percutaneous coronary intervention (PPCI) or thrombolytic therapy. Currently, timely and effective reperfusion of the myocardium is considered an important strategy to save viable myocardium, preserve the systolic function of the left ventricle and forestall heart failure [2]. Unfortunately, it is widely acknowledged that reperfusion can cause additional damage secondary to ischemic injury, i.e., I/R [3]. Mechanistically, I/R injury exacerbates myocardial tissue necrosis primarily through extensive inflammation and oxidative stress [4]. Recent research has provided evidence supporting the critical functions of pyroptosis in myocardial ischemia-reperfusion injury.

Pyroptosis, a specific mode of programmed death, relies on caspase-1 for its functioning [5]. As protein-mediated programmed necrosis, pyroptosis can release numerous intracellular pathogens and proinflammatory mediators through its unique membrane perforation mechanism [6,7]. Recently, studies have demonstrated that the cleavage of GSDMD protein via caspase-1/4/5/11 is the key point of pyroptosis death [6]. The N-terminal fragment is repositioned and inserted into the cell membrane, forming 10–14 nm pores and causing the leakage of IL-1β and IL-18, which will ultimately release cytosolic content [6,7]. The inflammatory response can lead to a series of abnormal tissue damage in repair disorders, thus promoting myocardial infarction (INF) area enlargement and adverse remodeling [8], whereas inhibiting the NLRP3 inflammasome pathway can alleviate inflammatory damage and reduce the INF area [9]. Therefore, pyroptosis can provide us with new strategies for myocardial ischemia-reperfusion injury.

Oridonin, a tetracycline diterpene derived from the Rabdosia rubescens, has been demonstrated to possess antitumor properties [10]. Recently, numerous studies have showed that oridonin exhibits good anti-inflammatory activities [11]. Research shows that oridonin inhibits glial cell activation and reduces inflammatory cytokine release in the hippocampus of amyloid-induced AD mice [12]. Liu et al. found that oridonin could ameliorate carbon tetrachloride-mediated liver fibrosis by regulating the NLRP3 inflammasome in mice [13]. However, it remains unknown whether oridonin can ameliorate myocardial I/R injury by modulating GSDMD-mediated pyroptosis. Thus, more investigations are warranted to address this question.

Our study was designed to elucidate the crucial role of oridonin during myocardial ischemia-reperfusion injury and uncover its underlying molecular mechanism via the regulation of GSDMD-mediated pyroptosis.

## 2. Materials and Methods

### 2.1. Isolation and H/R Treatment of Neonatal Rat Cardiomyocytes (NRCMs)

The hearts, which were isolated from 1- to 3-day-old Sprague–Dawley rats, were minced and added to the digestive solution (containing 0.06% type IV collagenase, 0.08% trypsin, 136 mM sodium chloride, 8.3 mM HEPES, 5.5 mM glucose, 4.2 mM sodium bicarbonate and 4 mM potassium chloride). The solution was then slowly stirred with a magnetic stirrer for 10 min at 37 °C and allowed to stand for 30 s. Supernatants were collected after solid digestion residues were skimmed off. Afterwards, we added 10 mL more digestion solution to the remaining tissue and stirred it at 37 °C for 10 min. Then, we repeated the above process 12 times. Subsequently, the lysates were centrifuged at 1000 rpm for 10 min, resulting in precipitation with a mixture of NRCMs and fibroblasts. The resuspended precipitated cells were cultivated with DMEM (Thermo Fisher Scientific, Waltham, MA, USA) containing 10% fetal bovine serum (Thermo Fisher Scientific, Waltham, MA, USA), 1% penicillin/streptomycin (pen/streptomycin, 10,000 U/mL each; Thermo Fisher Scientific, Waltham, MA, USA), and BRDU (Solarbio, Beijing, China) solution. This was then followed by the incubation of the cells at 37 °C for 1 h. These original cardiomyocytes were nonadherent and were harvested and cultured for subsequent experiments. The DMEM, supplemented with 4.5 g/L glucose, 10% (*v*/*v*) fetal bovine serum and 1% penicillin/streptomycin, was used to culture NRCMs in a 5% CO_2_ humidified incubator at 37 °C. After 1 h of exposure to 2.5 μM VX-765 (S2228, Selleck, Houston, TX, USA), 2.5 μM MCC950 (S8930, Selleck, TX, USA), and 2.5 μM Bay11-7082 (S2913, Selleck, Houston, TX, USA), cells in the different drug treatment groups were treated with 2.5 μM oridonin (O111381, Aladdin, Shanghai, China) for another 1 h before H/R treatment. The cardiomyocytes were exposed to hypoxic conditions (INVIVO2 500 O_2_ hypoxia cabinet: 1% O_2_, 5% CO_2_, and 94% N_2_) for 2 h, followed by normoxia for 4 h. We kept the control group under normoxic conditions (21% O_2_, 5% CO_2_, and 74% N_2_) for 6 h. All groups were cultured in FBS-containing DMEM (glucose-free) medium.

### 2.2. Mice and I/R Treatment

C57BL/6 mice (aged 6–8 weeks, weighing approximately 20–22 g) were obtained from the Wenzhou Medical University Animal Center and maintained under pathogen-free conditions. Our research was approved by the Animal Experiment Center of the First Affiliated Hospital of Wenzhou Medical University (ethics number WYYY-AEC-2022-010). The animals were reared in a 12-h/12-h light-dark cycle with free access to food and water. All procedures for the experiments were performed in strict compliance with the guidelines outlined in the National Institutes of Health Guide for the Care and Use of Animals. Thirty-two C57BL/6 mice were randomly allotted into 4 groups (control, control + oridonin, I/R, I/R + oridonin). Oridonin (15 mg/kg) or NaCl (0.9%) was injected intraperitoneally 2 h before I/R treatment, followed by peritoneal anesthesia with 1% pentobarbital sodium saline solution and endotracheal intubation into a small animal ventilator. The chest was opened on the left fourth rib. When the pericardium was torn, the left anterior descending (LAD) coronary artery was exposed. A 6–0 nonabsorbable thread (Surgical Specialties Corporation, Westwood, MA, USA) was used to ligate for 30 min at approximately 2–3 mm below the left auricle of the heart, followed by release and reperfusion for 2 h. The control group and the oridonin group had only an open chest and a ligation line but no ligation on the LAD.

### 2.3. Evans Blue/TTC Staining

The area of risk/left ventricle (AAR/LV) reflects the level of myocardial I/R injury, whereas the infarct size/area at risk (INF/AAR) represents the degree of myocardial death. The sutures under the LAD were briefly reoccluded after I/R surgery. To determine the infarct size, we went through the inferior vena cava and perfused animals with 2 mL 2% Evans blue (Sigma-Aldrich Company, St. Louis, MO, USA). Hearts, isolated and exposed 3 times to bleach with 10% PBS solution, were frozen at −80 °C for the following experiment: they were horizontally cut into five pieces (approximately 1 mm thick) from the tip and incubated with 1% 2, 3, 5-triphenyltetrachloride solution (TTC) in PBS at 37 °C for 15 min. After these procedures were completed, the heart was fixed with 10% formalin for 2 h. Images were analyzed by ImageJ software. The ratio of INF in AAR was counted using weight-based methods.

### 2.4. CCK-8 Assay

NRCMs were cultured in 96-well plates (containing 5000 cells per well) and divided into 8 groups: control and different concentrations. After each treatment, the medium was changed to 100 mL CCK-8 solution (containing 90 mL serum-free DMEM and 10 mL CCK-8 reagent) per well. Following the instructions from manufacturers (C0038; Beyotime Biotechnology, Shanghai, China), the viability of the cells was detected by visualizing the absorbance at 450 nm for each well for changes in color intensity.

### 2.5. Western Blot Analysis

Protein lysates were added to lyse mouse heart tissues and myocardial cells. The supernatant in different groups was gathered after centrifugation (12,000 rpm for 15 min). The proteins were split by SDS–PAGE before they were devolved into PVDF membranes. After the transfer, we blockaded the membrane with 5% skim milk for 1 h at 37 °C. The primary antibodies against NLRP3 (NBP2-12446, 1:1000; Novus Biologicals, Littleton, CO, USA), caspase-1 (sc-56036, 1:200; Santa Cruz Biotechnology, Dallas, TX, USA), and GSDMD (sc-393581, 1:200; Santa Cruz Biotechnology, Dallas, TX, USA) were washed with TBST solution and blocked overnight at 4 °C. The excess antibody was washed thoroughly with TBST solution 3 times for 10 min. The immunoreactive bands of the secondary antibody to goat anti-rabbit (A0208, 1:2000; Beyotime Biotechnology) and goat anti-mouse (A0216, 1:2000; Beyotime Biotechnology) with the corresponding bound secondary antibody were then visualized with ECL chemiluminescence and quantified by image software. GAPDH, a reference quantity, was used to qualify the protein expression.

### 2.6. Hematoxylin and Eosin (H&E) Staining

Samples from the hearts in each group were fixed with 4% paraformaldehyde and dehydrated with hierarchical alcohol (including 100%, 90%, and 70%). After treatment with xylene, the tissues were embedded in paraffin. These tissue specimens from all groups were sliced into 5 μm sections. After rehydration, hematoxylin-eosin staining was performed. These stained sections were examined by microscopy (Olympus Corporation, Tokyo, Japan).

### 2.7. Enzyme-Linked Immunosorbent Assay

The samples of cell supernatant and serum were collected. After that, IL-1β and IL-18 concentrations were determined with the protocol in the ELISA kit (Shanghai Xitang Technology Co, LTD, Shanghai, China). Absorbance of each sample was determined at 450 nm.

### 2.8. Immunohistochemistry

Paraffin sections of embedded myocardial tissue were dewaxed and rehydrated. After 60 min, the slices were dewaxed with xylene and then rehydrated with ethanol. Antigen retrieval was executed by microwave heating with sodium citrate solution for more than 15 min. After that, sections were blocked with 5% BSA and then incubated with an anti-GSDMD antibody (ab219800, 1:50, Abcam, Cambridge, MA, USA) overnight in a humidified chamber at 4 °C, followed by a corresponding secondary antibody (A0208, 1:200; Beyotime Biotechnology) at 20–37 °C for 1 h. Sections were incubated with diaminobenzidine and stained with hematoxylin after chromogenic staining. The images were reversed to blue with a hydrochloric acid alcohol differentiation solution, dehydrated again, sealed with neutral glue, and observed and collected under a microscope.

### 2.9. TdT-Mediated DUTP Nick End Labeling (TUNEL) Assay

These myocardial tissue paraffin sections were rehydrated in a gradient of xylene and ethanol following the kit instructions. Proteinase K solution was added dropwise for 30 min at 20–37 °C temperature. After 3 rinses with PBS for 10 min, the H_2_O was drained, and DAPI was added with an anti-fluorescence quenching seal. Images were observed and obtained under a confocal fluorescence microscope.

### 2.10. Immunofluorescence

The plates were washed in PBS, fixed for 10 min with 4% paraformaldehyde (pH 7.4), and permeabilized for 20 min with 0.5% Triton X-100 at room temperature. After washing with PBS, 5% FBS was added to the center of the dish and allowed to stand at 20–37 °C temperature for 30 min. The blocking solution was then removed, a sufficient dilution of primary antibody p65 (cst-8242s, 1:200; Cell Signaling Technology, Danvers, MA, USA) was added, and the cells were incubated at 4 °C overnight. A fluorescence-conjugated secondary antibody was added. After washing with PBST, the excess liquid was absorbed by absorbent paper with diluted fluorescent secondary antibody, incubated at 20–37 °C for 1 h, and soaked in PBS for 3 min each time. These steps were performed in the dark as much as possible. Finally, nuclei were counterstained with an anti-fluorescence quencher containing DAPI, and images were taken via a fluorescence microscope.

### 2.11. Statistical Analysis

All the experiments in groups involved in this study were repeated a minimum of 3 times. The data presented are the mean ± SD obtained from GraphPad Prism 8 (GraphPad, San Diego, CA, USA). We identified mean differences between groups via a one-way ANOVA with Tukey’s correction for multiple comparisons.

## 3. Results

### 3.1. Oridonin Reduced Infarct Size and Improved the Morphology of Cardiomyocytes

**Figure 1:**
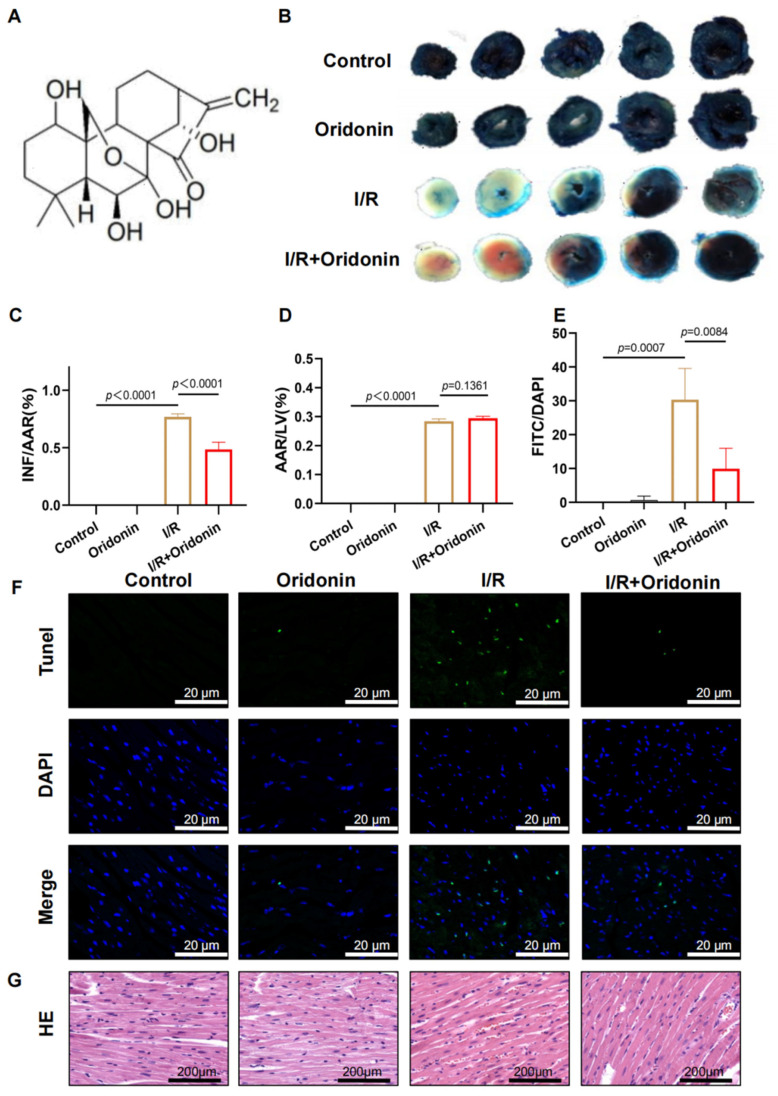
Oridonin reduced infarct size and improved the morphology of cardiomyocytes. (**A**) Chemical formula of oridonin. (**B**) Cross-sections of mouse left ventricles (LV) stained with Evans Blue/TTC. (**C**) Histogram of the INF/AAR results in (**B**). (**D**) Histogram of the AAR/LV results in (**B**). (**E**) Histogram of the number of TUNEL-positive cells based on (**F**). (**F**) Apoptosis of tissue cells was observed by TUNEL staining. The I/R group exhibited significantly higher cardiomyocyte pyroptosis (by approximately 30%) than the control group, and the number of TUNEL-positive cells was significantly reduced after oridonin pretreatment. (**G**) Representative H&E staining. The dose of oridonin pretreatment was 15 mg/kg. All samples were obtained from C57BL/6 mice. In all cases, the data are expressed as the mean ± SD.

The chemical structural formula of oridonin is shown in Figure 1A. To assess the impact of oridonin against myocardial ischemia–reperfusion damage, we measured the INF, AAR, and LV areas of C57BL/6 mice, and the results of AAR/LV revealed no distinct differences between the I/R group and the I/R + oridonin group. However, oridonin pretreatment significantly reduced the INF/AAR ratio in I/R. Compared with the I/R group, oridonin pretreatment (15 mg/kg) effectively protected against I/R injury in the heart tissue (Figure 1B–D). Moreover, the I/R group performed markedly more (approximately 30%) apoptotic cardiomyocytes than the control group. Oridonin pretreatment significantly decreased the number of apoptotic cardiomyocytes after ischemia-reperfusion injury during I/R (Figure 1E,F). Changes in cardiomyocyte morphology were detected by H&E staining. Besides, in the control group, cardiomyocytes were morphologically holonomic and neatly arranged. In stark contrast, cells were disordered, swollen and characterized by nuclear and cytoplasmic dispersion, myofibril contracture and sarcolemma disruption in the I/R group. In the I/R + oridonin (15 mg/kg) group, however, the quantity of swollen cardiomyocytes was significantly reduced and there were fewer morphological changes (Figure 1G).

### 3.2. Oridonin Reduces IR-Induced Myocardial Pyroptosis In Vivo

**Figure 2:**
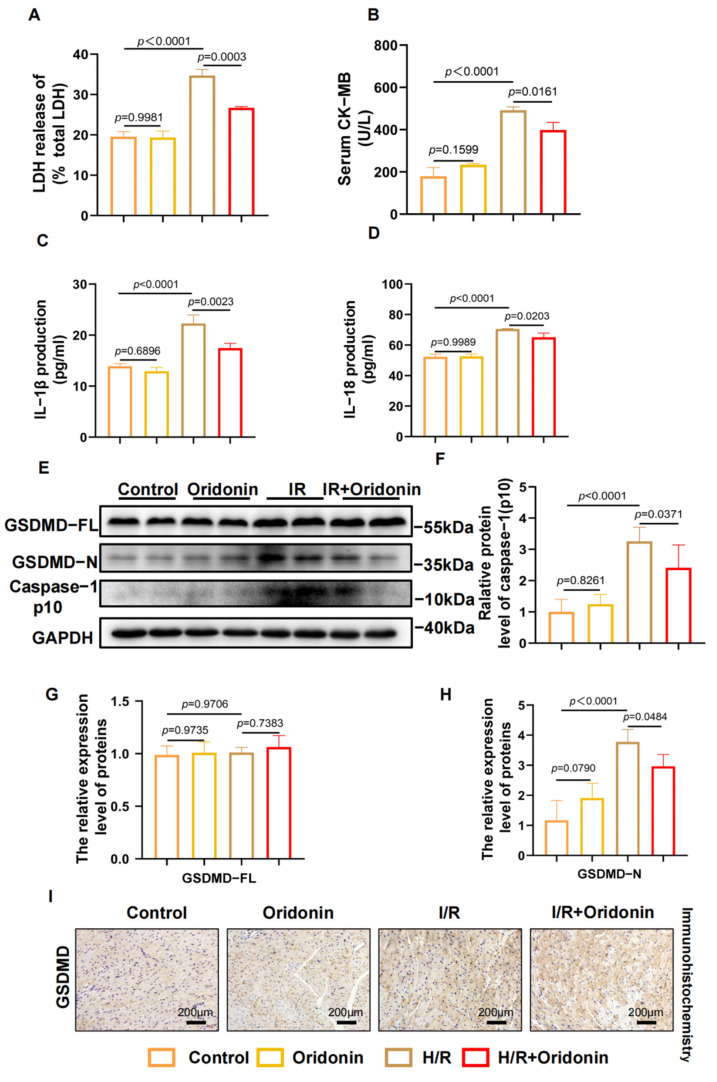
Oridonin reduces IR-induced myocardial pyroptosis in vivo. (**A**) The LDH content in the serum from each group. (**B**) The content of CK-MB in serum from each group. (**C**) The content of IL-1β in serum from each group. (**D**) The content of IL-18 in serum from each group. (**E**) Typical western blot of GSDMD-FL, GSDMD-N, and cleave-caspase-1 from each group. (**F**) Densitometric quantification of cleave-caspase-1 based on (**E**). (**G**) Densitometric quantification of GSDMD-FL based on (**E**). (**H**) Densitometric quantification of GSDMD-N based on (**E**). (**I**) Representative immunohistochemistry results from each group. The protein level was standardized using GAPDH. All samples were obtained from C57BL/6 mice. In all cases, the data are expressed as the mean ± SD.

Although our research demonstrated that oridonin pretreatment can ameliorate I/R injury in vivo in C57BL/6 mice, we have yet to uncover its underlying mechanism. We hypothesized that the protective mechanism of oridonin is related to GSDMD-mediated pyroptosis. Measurements of LDH and CK-MB in serum revealed that I/R infarction resulted in significant myocardial injury in C57BL/6 mice, and pretreatment with oridonin prevented I/R injury (Figure 2A,B). This was also corroborated by the ELISA results of animal serum for the cytokines IL-1β and IL-18 (Figure 2C,D). After I/R treatment, the expression of GSDMD-N and cleave-caspase-1 was increased in left ventricular tissue, but the expression of GSDMD-FL was not increased. In addition, we found that the expression levels of GSDMD-N and cleaved caspase-1 were notably decreased in the I/R + oridonin group compared with those of the I/R group (Figure 2E–H). The immunohistochemical results of GSDMD in paraffin sections were congruent with those of western blot analysis (Figure 2I). Collectively, our results show that oridonin ameliorates myocardial ischemia-reperfusion injury by affecting GSDMD-mediated pyroptosis.

### 3.3. Oridonin Protected Cardiomyocytes from H/R-Induced Pyroptosis

**Figure 3:**
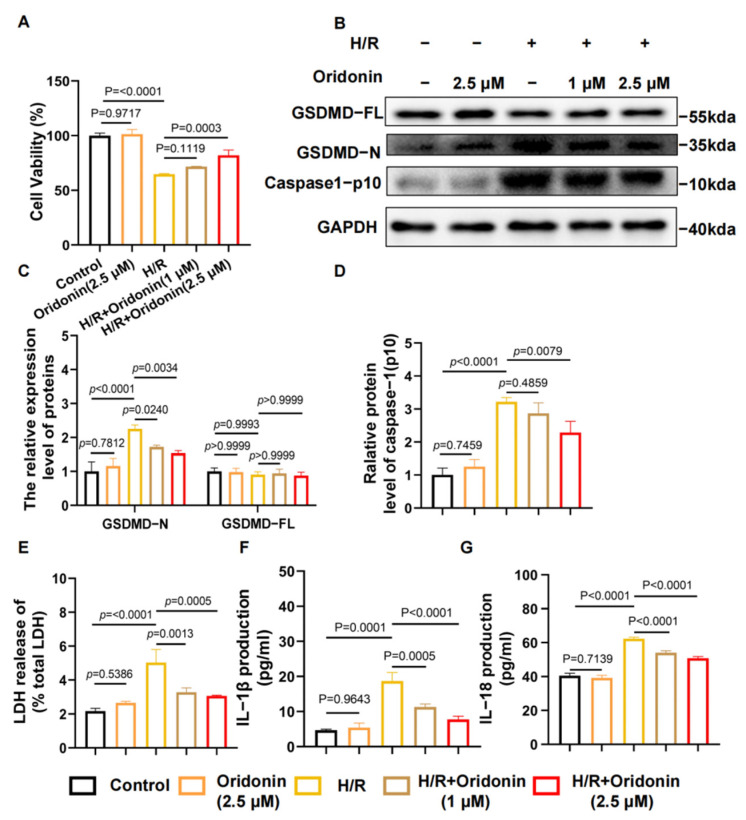
Oridonin protected cardiomyocytes from H/R-induced pyroptosis. (**A**) Cell viability of NRCMs from each group. (**B**) Representative western blot results of GSDMD-FL, GSDMD-N and cleave-caspase-1 from each group. (**C**) Densitometric quantification of GSDMD-N and GSDMD-FL based on (**B**). (**D**) Densitometric quantification of cleave-caspase-1 based on (**B**). (**E**) Percent LDH release in cell supernatants from each group. (**F**) Concentration of IL-1β in the cell supernatants from each group. (**G**) Concentration of IL-18 in the cell supernatants from each group. All samples are from NRCMs. The protein level was standardized using GAPDH. In all cases, the data are expressed as the mean ± SD.

Although we demonstrated that oridonin pretreatment can protect the myocardial tissue of mice after I/R treatment by inhibiting pyroptosis, we wondered if this mechanism also applies to NRCMs under H/R treatment. To identify the optimal concentration of oridonin treatment, we constructed 1-h pretreatments with concentrations of 0–80 μM. Although there was no statistically significant effect of 5 μM treatment on cell viability, there was a trend toward decreased viability (Supplementary Figure S1A). Thus, we selected 1 and 2.5 μM for the subsequent experiment. To demonstrate that oridonin mainly acts on NRCMs, we also established a primary fibroblast model in Sprague–Dawley rats for auxiliary verification. Microscopically, we observed that pretreatment with oridonin improved the morphology and viability of H/R-exposed rats but had no protective effect on H/R-exposed primary fibroblast damage, i.e., oridonin did not affect the activity of rat primary fibroblasts under H/R but only maintained the activity of NRCMs (Supplementary Figure S1B). Following exposure to H/R, the content of GSDMD-N and cleaved caspase-1 were significantly increased in NRCMs, and oridonin markedly reduced the content of these proteins in NRCMs. The cell viability results were also consistent with this trend. However, the concentration gradients revealed no significant difference in GSDMD-FL expression (Figure 3A–D). The content of IL-1β, IL-18 and LDH in the supernatants was consistent with the western blot results (Figure 3E–G).

### 3.4. Oridonin Regulates GSDMD-Mediated Pyroptosis via the NF-κB/NLRP3 Signaling Pathway

**Figure 4:**
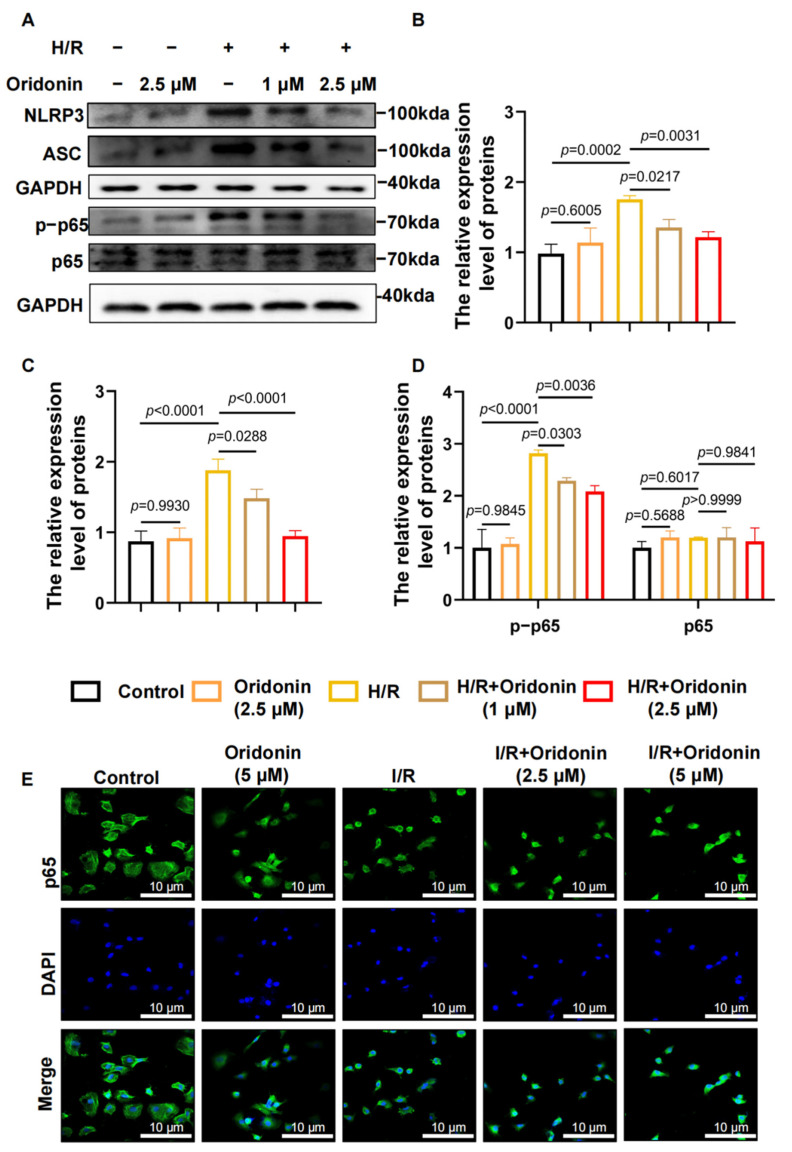
Oridonin participates in the regulation of the NF-κB/NLRP3 signaling pathway. (**A**) Representative western blot results of NLRP3, ASC, p65, and p-p65 from each group. (**B**) Densitometric quantification of NLRP3 based on (**A**). (**C**) Densitometric quantification of ASC based on (**A**). (**D**) Densitometric quantification of the ratio of p65/p-p65 based on (**A**). (**E**) Representative immunohistochemistry results in NRCMs from each group. All samples are from NRCMs. The protein level was standardized using GAPDH. In all cases, the data are expressed as the mean ± SD.

Activation of the NLRP3 inflammasome is a key step in exacerbated pyroptosis in myocardial ischemia-reperfusion injury, and inhibition of NLRP3 inflammasome formation can effectively limit myocardial injury after myocardial reperfusion in mice [14]. As a key factor in inflammation [15], p65 can stimulate the transcription of downstream inflammatory proteins after IκB phosphorylation [16,17]. Thus, we tracked the expression of ASC, NLRP3, p65, and p-p65. The results suggested that related proteins in the H/R group had higher expression than those in the control group, whereas oridonin pretreatment suppressed the expression of ASC, p-p65 and NLRP3. There was no statistically significant difference in p65 expression levels among the groups. This finding suggests that oridonin could inhibit the phosphorylation of p65 during H/R and suppress the formation of the NLRP3 inflammasome (Figure 4A–D). In addition, we also monitored the expression of p65 in NRCMs during H/R by immunohistochemical fluorescence. We demonstrated that p65 was concentrated in the nucleus in the H/R group and dispersed in the cytoplasm in the H/R + oridonin group, suggesting that p65 was activated during H/R treatment and suppressed by oridonin, a finding that was also consistent with the western blot results (Figure 4E).

**Figure 5:**
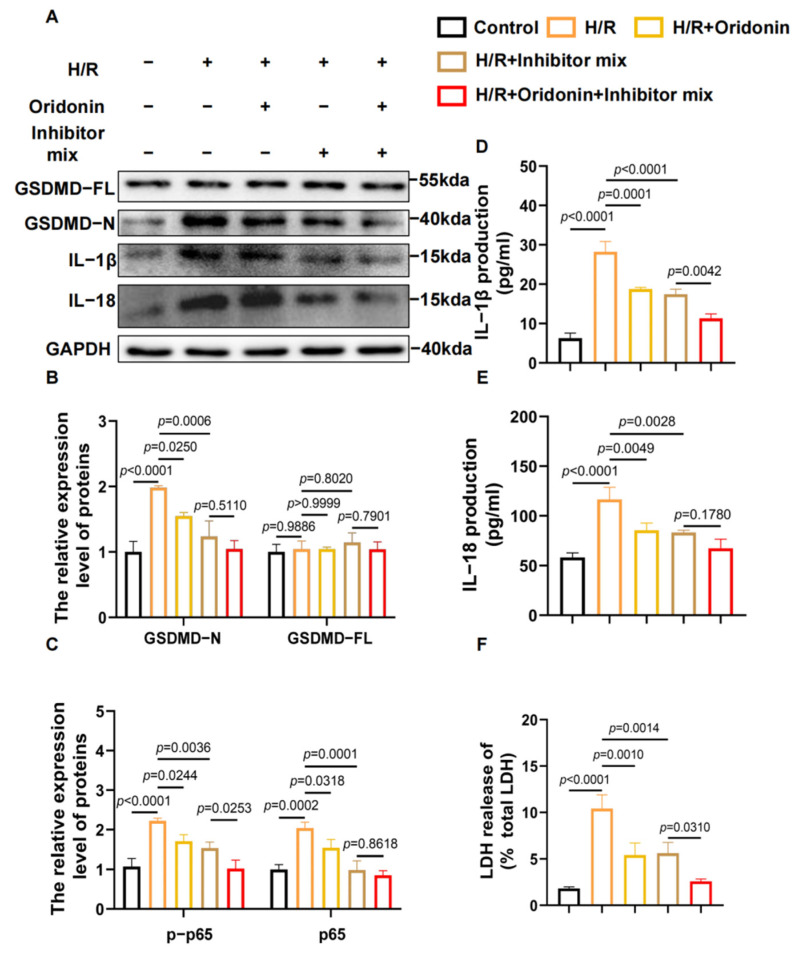
Oridonin regulated GSDMD-mediated cell pyroptosis via the NF-κB/NLRP3 signaling pathway. (**A**) Representative western blot results of GSDMD-FL, GSDMD-N, IL-1β and IL-18 from each group. (**B**) Densitometric quantification of GSDMD-N and GSDMD-FL based on (**A**). (**C**) Analysis of the densitometric measurements of IL-1β and IL-18 based on (**A**). (**D**) Concentrations of IL-1β in cell supernatants from each group. (**E**) Concentrations of IL-18 in cell supernatants from each group. (**F**) Concentrations of LDH in cell supernatants from each group. The protein level was standardized using GAPDH. The mixed inhibitor contained 5 μM Bay-117082 (NF-κB inhibitor), MCC950 (NLRP3 inhibitor) and VX-765 (caspase-1 inhibitor). All samples are from NRCMs. In all cases, the data are expressed as the mean ± SD.

To elucidate the pathway by which oridonin affects the NLRP3 inflammasome, we pretreated NRCMs with a mixed inhibitor containing 5 μM Bay-117082 (NF-κB inhibitor), MCC950 (NLRP3 inhibitor) and VX-765 (caspase-1 inhibitor) for 1 h before H/R. The expressions of GSDMD-N, IL-1β, and IL-18 were inhibited after H/R in the presence of the inhibitors, and the related pyroptosis protein was further downregulated in the H/R module along with oridonin, suggesting that oridonin may further reduce the damage via the NLRP3 and NF-κB pathways (Figure 5A–C). Furthermore, the determinations of LDH, IL-1β and IL-18 from the cell supernatants also support this conclusion (Figure 5D–F).

## 4. Discussion

In our study, GSDMD-mediated pyroptosis is the main mechanism explored in myocardial ischemia-reperfusion injury. First, we verified that GSDMD was activated when mice developed ischemia-reperfusion injury. Previous research has already established that oridonin can specifically bind to the hemi-frame amino acid at position 279 of the NACHT domain to form a covalent bond that blocks the NLRP3 and NEK7 interaction, leading to the inhibition of the assembly and activation of NLRP3-mediated inflammasome [18]. Building on this finding, we pretreated with oridonin to confirm its effect on the NF-κB/NLRP3 inflammasome pathway and the characteristics of the downstream pyroptosis pathway in vitro and in vivo. Recently, many studies of signaling pathway-based drugs have provided evidence in favor of the efficacy of molecularly targeted therapy. As a promising therapeutic strategy, this technique has also revolutionized cancer treatment and significantly improved patient outcomes and five-year survival rates [19]. As a targeted inhibitor of NLRP3, oridonin has also opened up new ways of thinking for the remedy of acute myocardial ischemia-reperfusion injury.

When Toll-like receptors are activated under stress conditions such as hypoxia, various messenger proteins are recruited. These molecules activate NF-κB to exert specific effects on the corresponding stimuli. NLRP3 is a group of multiprotein complexes of the inflammasome that are primarily triggered by infection or tissue damage. When NLRP3 is activated, it can specifically exert its biochemical function, cleaving pro-caspase-1 and generating activated caspase-1 [20]. The fragment of GSDMD-N oligomerizes with phosphatidylserine and cardiolipin on cell membranes and forms pores in liposomes that mediate the cellular release of packaged mature IL-1β [21,22]. As a specific inhibitor of the NLRP3-mediated inflammasome, AER was demonstrated to be effective as a covalent inhibitor capable of reducing upstream inflammatory cytokine triggering. Currently, there is mounting evidence that oridonin can inhibit I/R injury, including injury to the myocardium, lower limbs, brain tissue, and kidney, by downregulating oxidative stress and NLRP3-mediated inflammatory pathways [9,23,24]. Although these studies have included metabolomics, exosomes, Mincle, and NF-κB, none of the mechanisms have clarified the key role of GSDMD-mediated pyroptosis [25,26,27]. In our study, treatment with H/R and oridonin did not alter the expression of GSDMD-FL. In contrast, oridonin treatment decreased the relative expression of GSDMD-N. In addition, the expression of ASC, p-p65 and the NLRP3 inflammasome was increased after H/R treatment, further confirming the findings of previous studies. Thus, the NF-κB and NLRP3 inflammasomes appear to be at least partially responsible for pyroptosis when I/R damage occurs. By suppressing the expression of NF-κB and NLRP3 inflammasomes, oridonin can reduce the incidence of GSDMD-N, thereby attenuating ischemia-reperfusion injury of cardiomyocytes due to the activation of pyroptosis.

**Figure 6:**
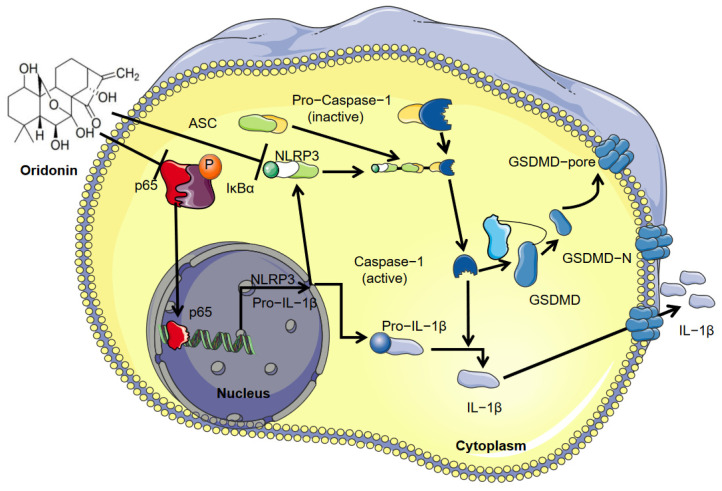
A schematic representation of the protective effect of oridonin during ischemia-reperfusion injury in the heart by inhibiting GSDMD-mediated pyroptosis.

In our study, we offered experimental evidence that oridonin can inhibit GSDMD through NF-κB/NLRP3 pathway-mediated pyroptosis, thereby alleviating myocardial I/R injury (Figure 6). As a result, oridonin has the potential to be used as a novel medicine for the prevention of myocardial I/R injury in clinical settings. In addition, our findings also provide novel thinking for the application of the NF-κB/NLRP3 inflammasome pathway in myocardial I/R injury. We hope that our findings can provide some experimental evidence to support the potential application of oridonin in clinical settings in the future.

## Data Availability

Data will be made available on request.

## Supplementary Materials

The following supporting information can be downloaded at: https://www.mdpi.com/article/10.3390/genes13112133/s1, Supplemental Figure S1: Oridonin reduced cardiomyocyte damage but not fibroblast damage during cardiac infarction. (A) The safe concentration of oridonin in NRCMs. (B) Images of NRCMs and primary fibroblasts from each group. All samples were obtained from Sprague–Dawley rats. In all cases, the data are expressed as the mean ± SD.

## Abbreviations

H/R	hypoxia/reoxygenation
I/R	ischemia/reperfusion
NRCMs	neonatal rat cardiomyocytes
CCK	cell counting kit
TUNEL	terminal-deoxynucleotidyl transferase-mediated nick end labeling
INF	infarct size
AAR	area at risk
LV	left ventricle
Ori	oridonin
LDH	lactate dehydrogenase
CK-MB	creatine kinase isoenzyme
GSDMD-FL	gasdermin D-full length
GSDMD-N	gasdermin D-N domain
