# Supplementary material for: Oridonin Protects against Myocardial Ischemia–Reperfusion Injury by Inhibiting GSDMD-Mediated Pyroptosis
Source: Genes (Basel). 2022 Nov 17;13(11):2133. doi: 10.3390/genes13112133 (PMC9690185; doi:10.3390/genes13112133)
Supplement: Supplementary file 1 [file genes-13-02133-s001.zip › genes-1987212-SI.pdf]

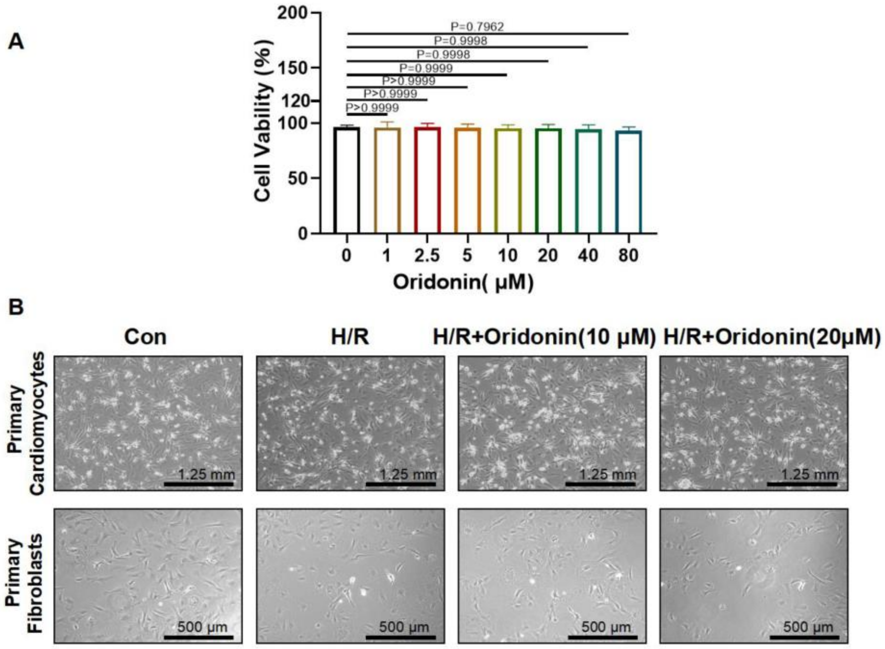

Figure S1: Oridonin reduced cardiomyocyte damage but not fibroblast damage during cardiac infarction. (**A**) The safe concentration of oridonin in NRCMs. (**B**) Images of NRCMs and primary fibroblasts from each group. All samples were obtained from Sprague–Dawley rats. In all cases, the data are expressed as the mean ± SD.
